# Supplementary material for: Novel Red Light-Absorbing Organic Dyes Based on Indolo[3,2-b]carbazole as the Donor Applied in Co-Sensitizer-Free Dye-Sensitized Solar Cells
Source: Materials (Basel). 2021 Mar 31;14(7):1716. doi: 10.3390/ma14071716 (PMC8037655; doi:10.3390/ma14071716)
Supplement: Supplementary file 1 [file materials-14-01716-s001.pdf]

materials

MDPI

Supplementary

Novel red light-absorbing organic dyes based on indolo[3,2-b]carbazole as the donor applied in co-sensitizer free dye-sensitized solar cells

Zhanhai Xiao [1,2], Bing Chen [2,*] and Xudong Cheng, [1,*]

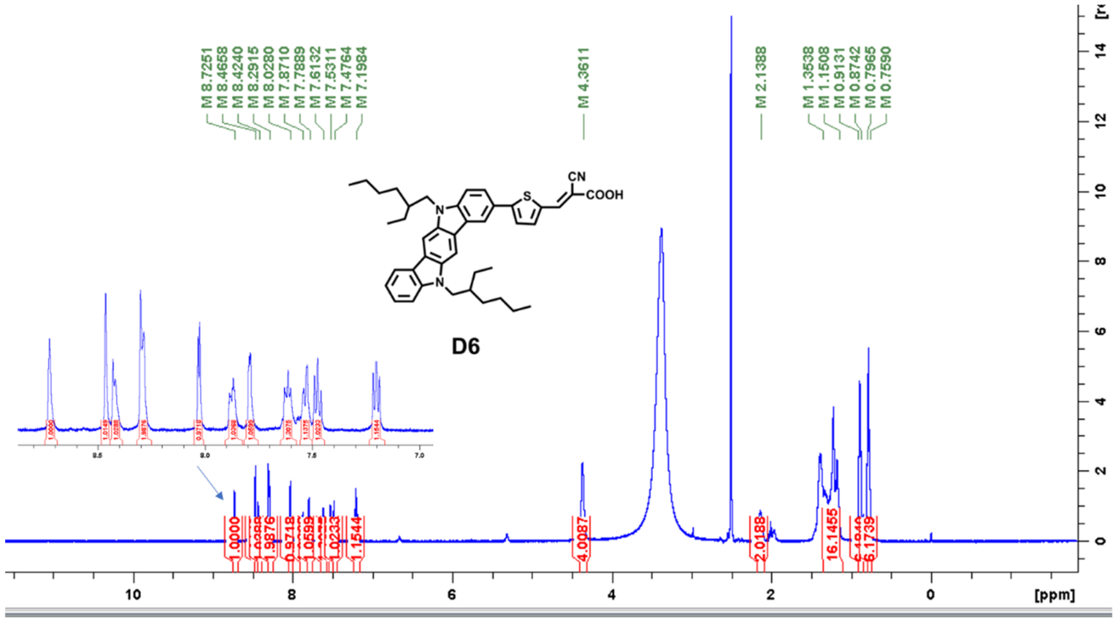

Figure S1. ^{1}H NMR of dye **D6** in DMSO-d6.

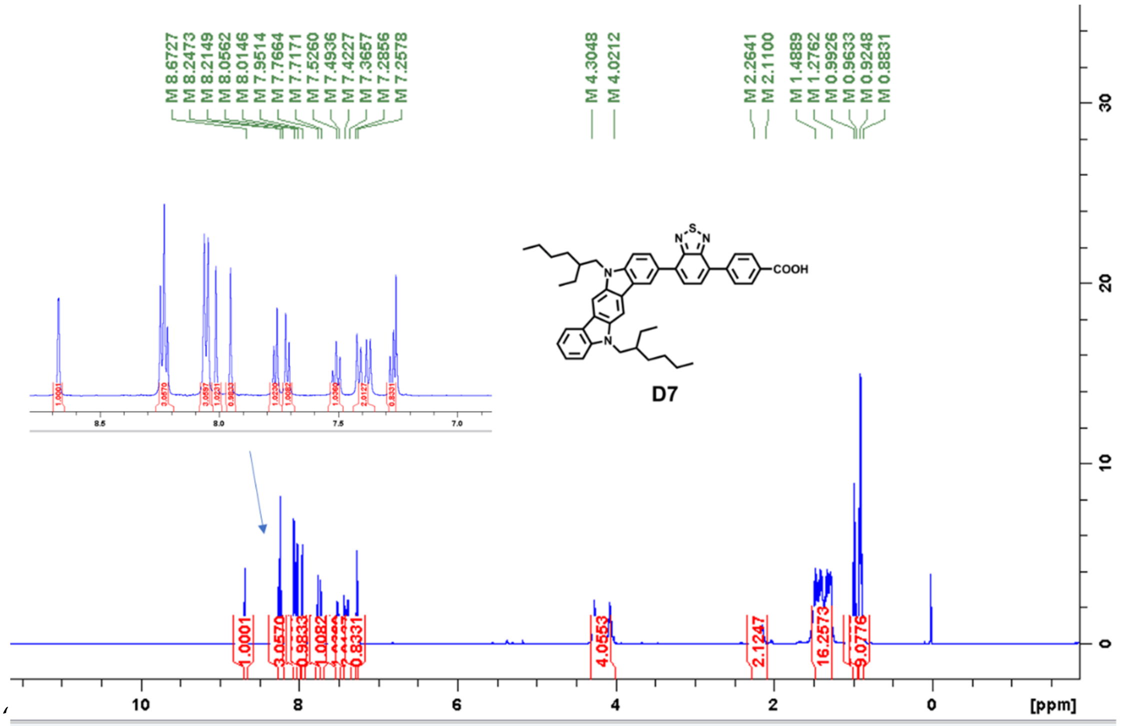

Figure S2. ^{1}H NMR of dye **D7** in $CDCl_3$.

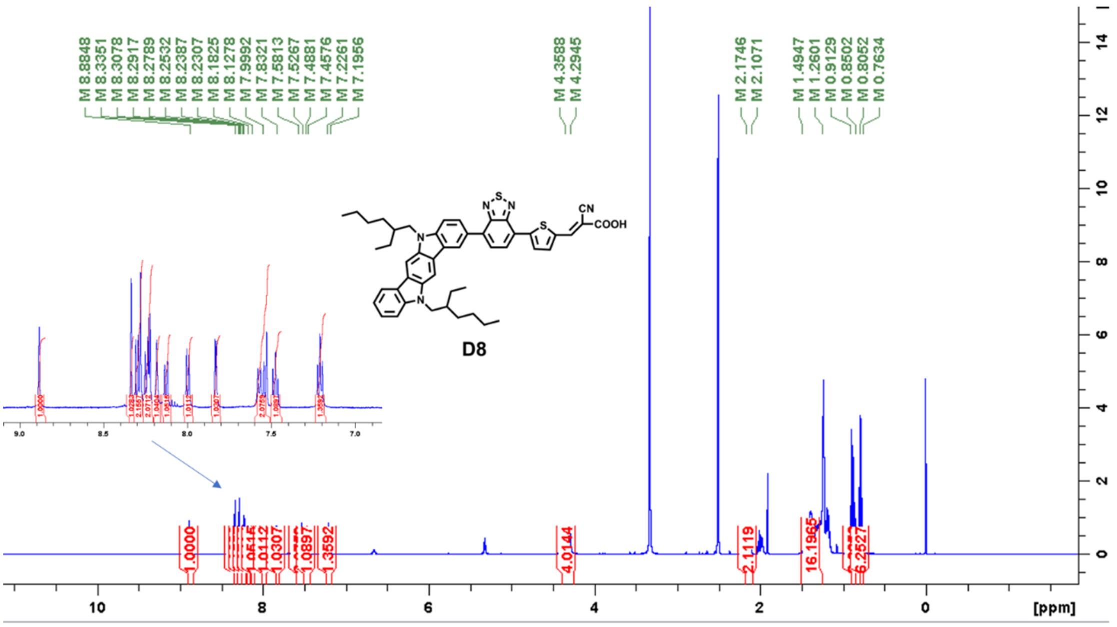

Figure S3. ^1H NMR of dye **D8** in DMSO-d6.

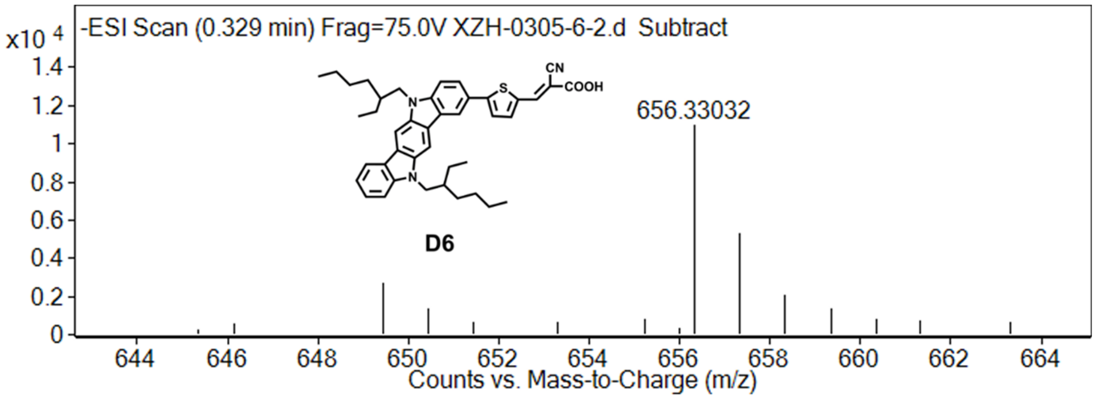

Figure S4. Mass spectra of dye **D6.**

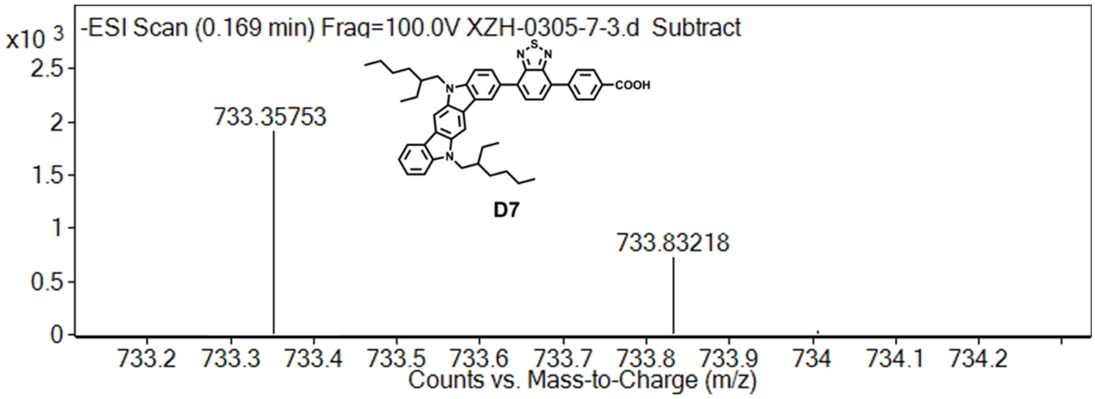

Figure S5. Mass spectra of dye **D7.**

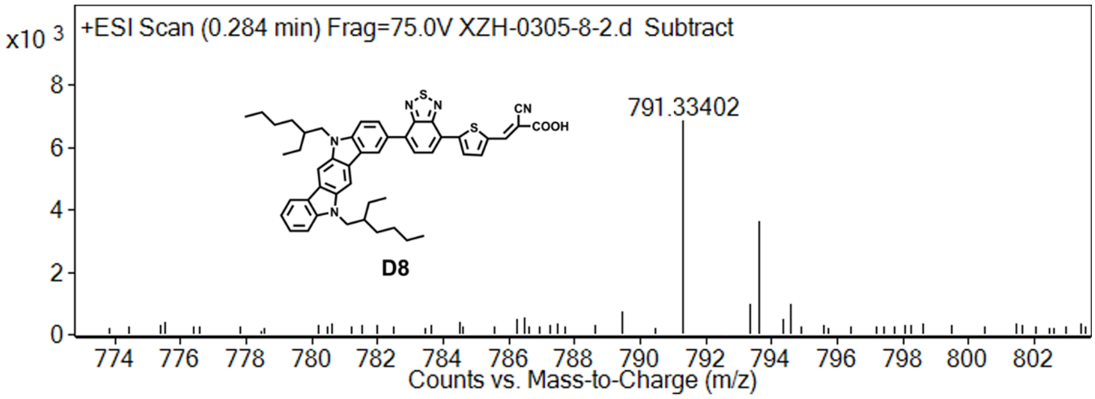

Figure S6. Mass spectra of dye **D8.**
